# Prevalence of anti-*Leptospira* antibodies and associated risk factors in the Malaysian refugee communities

**DOI:** 10.1186/s12879-021-06830-0

**Published:** 2021-11-01

**Authors:** Izzah Ruzana Mohd Hanapi, Norhidayu Sahimin, Michael John Banuru Maackara, Aufa Shofia Annisa, Raisya Nur Syazmeen Abdul Mutalib, John W. Lewis, Jerzy M. Behnke, Yee Ling Lau, Siti Nursheena Mohd Zain

**Affiliations:** 1 Institute of Biological Sciences, Faculty of Science, Universiti Malaya, 50603 Kuala Lumpur, Malaysia; 2 Tropical Infectious Diseases Research and Education Centre (TIDREC), Universiti Malaya, 50603 Kuala Lumpur, Malaysia; 3 Department of Biomedical Science, Kulliyah of Allied Health Science, International Islamic University Malaysia, 25200 Kuantan, Pahang Malaysia; 4 School of Biological Sciences, Royal Holloway, University of London, Egham, Surrey, TW20 OEX UK; 5 School of Life Sciences, University of Nottingham, University Park, Nottingham, NG7 2RD UK; 6 Department of Parasitology, Faculty of Medicine, Universiti Malaya, 50603 Kuala Lumpur, Malaysia

**Keywords:** Prevalence, Leptospirosis, Refugee, IgG, IgM, Malaysia

## Abstract

**Background:**

Refugees in Malaysia, who are afflicted by poverty, conflict and poor health, are vulnerable to a range of zoonotic infections in the deprived environmental and social conditions under which they live. Exposure to infections such as leptospirosis, for which rodents are primary hosts, is of particular concern.

**Methods:**

A wellness program was conducted to determine the presence of antibodies against *Leptospira* (seroprevalence) in 11 refugee community schools and centers in the Klang Valley, Malaysia. A total of 433 samples were assessed for IgG and IgM antibodies against *Leptospira*, using enzyme-linked immunosorbent assays (ELISA).

**Results:**

Overall *Leptospira* seroprevalence was 24.7%, with 3.0% being seropositive for anti-*Leptospira* IgG and 21.7% for anti-*Leptospira* IgM. Factors significantly associated with overall *Leptospira* seroprevalence included: age, ethnicity, pet ownership, knowledge of disease and awareness of disease fatality. For IgM seroprevalence*,* significant risk factors included sex, ethnicity, eating habits with hands, pet ownership, the presence of rats, walking in bare feet and water recreation visits.

**Conclusions:**

These findings highlight the need for improvements in health and well-being among the refugee community through disease awareness programs and provision of healthy behavior programs, particularly in hygiene and sanitation through community engagement activities.

## Background

The United Nations High Commissioner for Refugees (UNHCR) has highlighted the limited access to basic services, such as healthcare, formal education and the right to work, among refugees in Malaysia [1]. The exclusion of these essential services for refugees stems primarily from Malaysia not being a party to the 1951 Refugee Convention and its 1967 protocol. Additional barriers include language and cultural differences, unaffordability, poor health literacy, social and protection issues [2]. Nevertheless, the influx of refugees and asylum-seekers entering this country over the last 5 years has increased. As of the end January 2021, there were 178,710 registered refugees and asylum-seekers in Peninsular Malaysia the majority of whom were from Myanmar (~ 154,140) comprising Rohingyas (66.4%), Chins (14.6%) and other sub-ethnic groups (19%). The remaining 24,560 (13.7%) originated from Pakistan, Yemen, Syria, Somalia, Afghanistan, Sri Lanka, Iraq, Palestine and other countries [3].

Refugees in Malaysia generally reside in low-cost flats in urban cities such as Kuala Lumpur and Penang and live amongst local communities instead of camps [4, 5]. Nevertheless, they experience heavy restrictions with respect to formal education, work and health care [6]. Therefore, the UNHCR in collaboration with local and international partners, including non-governmental and religious organizations, has introduced and supported community-based learning centers for refugee children. Up to 120 such centers exist in West Malaysia with foci in the Klang Valley, Johor and Penang but these only operate in vicinities with large numbers of refugees and have a range of 60 to 100 children in attendance. The majority of teachers are volunteers from the same communities and parents who appreciate that education is essential to ensure stability and normality of their children [6].

Leptospirosis is an endemic disease in Malaysia [7–11] and in 2010 the Ministry of Health declared leptospirosis to be a notifiable disease [12]. Data reported by the Disease Control Division of the Ministry of Health in Malaysia in the e-Notifikasi system, showed that the incidence of leptospirosis was also lower in 2017 compared with 2016 (4365 cases of leptospirosis including 27 deaths in 2017, compared to 5285 cases with 52 deaths in 2016). Among positive cases, 68% were men with 47% being 25 to 55 years old. There were 16 confirmed outbreaks of leptospirosis and most occurred in residential areas [13]. Refugees entering Malaysia through the maritime route or by land via the Thailand transit camp [14] are most likely exposed to the transmission of leptospirosis, as they experience poor social and environmental conditions, although a study recorded low seroprevalences among an urban poor population living in low-cost flats and squatter settlements [15].

However, the present study is the first in Malaysia to assess seroprevalence of leptospirosis among urban refugees, and to evaluate their health status and associated risk factors. This in turn will serve as a reference for stakeholders such as NGOs, encouraging and justifying the allocation of resources for improving the health and welfare of these refugee communities.

## Methods

### Enrolment of participants

This cross-sectional study was conducted from April 2019 to March 2020 in Klang Valley, one of the most active business areas in Kuala Lumpur, the capital city of Malaysia. The Klang Valley interconnects cities and towns from two States: Selangor and the Federal Territory of Kuala Lumpur where over 52.0% of 178,710 refugees and asylum seekers, who are registered in Peninsular Malaysia, reside with up to 66,030 (36.9%) in Selangor and 27,370 (15.3%) in Federal Territory of Kuala Lumpur [3]. Despite the presence of several modern houses and apartments, Klang Valley largely comprises shanty houses and high-rise low-cost flats, which are mainly inhabited by low-income families, migrants, refugees and asylum-seekers. Several refugee community schools are also located in these areas.

### Wellness program

This study was conducted through a social wellbeing community program. Apart from sample collection and interviews, physical screening such as body mass index (BMI) measurements, and blood pressure checkups were provided for all participants. Social wellbeing was promoted also through the provision of education talks on health issues. Free sanitary kits, including deworming tablets, were given to all participants.

### Estimation of sample size

Sample size was estimated to be 170 participants, based on a formular reported by Krejcie and Morgan [16], with the following input parameters: crude seroprevalence of 12.6% (based on a study on leptospirosis among urban poor in Kuala Lumpur [15]), with a 5% margin of error and 95% confidence intervals. Since recruitment was voluntary, we ended up with up to 433 refugees of all ages, sex and ethnicity, from the 11 community schools/centres. The number of refugees sampled from each school varied from eight to 99 (mean = 39.4), but since all schools/centres were located within close vicinity to one another, we have chosen to treat all refugees for analysis as one population.

### Data collection: blood samples and risk factors for *Leptospira* seroprevalence

Structured questionnaires were presented to all participants after consent had been given. Parents, guardians and/or heads of respective centres/schools completed the consent forms for those under 18 years of age. Face-to-face interviews were conducted in each location to assess the risks of exposure to leptospirosis. The questionnaire was divided into several sections according to socio-economic, demographic, sanitation and environmental indicators, together with background knowledge, aetiology and symptoms of leptospirosis. Questionnaires recorded education attainment, types of accommodation and lifestyle behaviours such as eating habits, sources of drinking water, appropriate waste disposal, frequency of hand washing and pet ownership. Refugees were also tested on their basic knowledge of leptospirosis such as mortality, disease transmission, aetiology, and risk factors for infection.

At the time of interview, 3–6 ml of venous blood were collected from each participant, into non-anticoagulant tubes and transported to the laboratory in an icebox within 2–4 h after collection. Samples were then centrifuged using a fixed-angle-rotor centrifuge (Universal 320 Centrifuge, *Hettich* Laboratory, Germany) at 2500–3000 rpm for 5–10 min. Sera were aliquoted into tightly capped polystyrene tubes and stored at − 20 °C.

### Serological analysis

The presence of anti-*Leptospira* antibodies was determined using Commercial ELISA kits (Virion/Serion ELISA classic *Leptospira* IgG/IgM) [17] purchased from UC Biosciences Sdn Bhd, Kuala Lumpur, Malaysia. The kits were kept at 2–8 °C and reagents and samples were diluted following recommendations in the Serion Diagnostic protocol version V 125.15. IgG and IgM optical densities (OD) were measured against a blank substrate at a wavelength of 405 nm using a TECAN ELISA plate reader (Tecan Austria GmbH, Austria). The sensitivity (94.7%) and specificity (> 99%) of the test kits were calculated by combining IgG and IgM data, without borderlines, of 63 sera from patients with suspected leptospirosis. Sera were compared with healthy blood donors using the serovar-specific complement fixation test (CFT) as the latter does not differentiate between IgG and IgM antibodies.

Positive and negative controls, a substrate blank and standard references, supplied with *Leptospira* IgG/IgM test kits were evaluated, subject to variation between kit/lot numbers. For *Leptospira* IgG (Lot: SMH.AX IFU-Version 125–15) OD values of < 10 U/ml were considered to represent negative samples, compared with intermediates/borderlines of 10–15 U/ml and positive values of > 15 U/ml, the latter indicating earlier exposure to infection and a host recovery phase. For *Leptospira* IgM (Lot: SEI.EB IFU-Version 125-15), OD values of < 15 U/ml were considered to be negative compared with intermediates/borderline values of 15–20 U/ml and positive values of > 20 U/ml confirming exposure to new, recent infections.

### Data analysis

All data were analysed using the SPSS software version 22. Summary data are provided for prevalence of infection (percentage infected in relevant factor levels) plus 95% confidence limits (95% CL) calculated by bespoke software based on the statistical tables of Rohlf and Sokal [18]. Odds ratios + 95% confidence limits were calculated for levels within each factor using one level as the reference point in each case. In both cases 95% confidence limits are given in tables and in the text 95% confidence intervals are illustrated in relevant figs.

Statistical analyses were conducted using maximum likelihood techniques based on log linear analysis of contingency tables in the software package IBM SPSS (vers. 22). This approach is based on categorical values of factors of interest, which are used to fit hierarchical log linear models to multidimensional cross-tabulations using an iterative proportional-fitting algorithm that detects associations between these factors, one of which is the presence/absence of antibody. First, exploratory models were fitted with the presence/absence of IgG or IgM relative to single explanatory factors of interest, i.e. sex (male, female), age class (< 12, 12–18 or > 18 years old) and ethnicity (Rohingya, Rakhine, Chin, Kachin, Pakistan and Syria). Other factors taken into account included educational attainment, types of accommodation and also lifestyle factors such as eating methods, sources of drinking water, appropriate waste disposal, frequency of hand washing and pet ownership. Refugees were also tested on their basic knowledge of leptospirosis such as mortality, disease transmission, etiology, contact with rats/rat urine and the awareness associated with walking in bare feet, flooding and visits to water recreation centres. After the initial round of exploratory analyses, multifactorial models were fitted incorporating only the significant factors identified in the first round of analysis. Following stepwise backward selection, multifactorial minimum sufficient models (MMSM) were generated, and outputs of these models are reported.

## Results

### Socio-demographic characteristics and seroprevalences of anti-*Leptospira* IgG and IgM antibodies

The study was conducted over a period of 1 year, from April 2019 to March 2020 in order to achieve the required sample size. A total of 433 refugees from 11 refugee learning centers participated in this study including Selayang (*n* = 99; 22.9%), Puchong (n = 63; 14.5%); Bukit Bintang (n = 60; 13.9%); Setapak (n = 49; 11.3%); Kajang (n = 34; 7.9%); Taman Setapak (n = 32; 7.4%); Pudu (n = 26; 6%); Cheras (n = 25; 5.8%); Bangi (n = 19; 4.4%); Kuchai Lama (n = 18; 4.2%) and Klang (n = 8; 1.8%). The socio-demographic profile comprised of 223 males (51.5%) and 210 females (48.5%). Among them, 34.9% were less than 12 years old (n = 151), 33.2% were above 18 years old (n = 144) and 31.9% were between 12 and 18 years old (n = 138). Most participants were comprised of Kachin ethnicity (n = 185, 42.7%), followed by Rohingya (n = 107, 24.7%), Rakhine (n = 63, 14.5%), Pakistan (n = 34, 7.9%), Chin (n = 25, 5.8%) and Syrian (n = 19, 4.4%). The majority of participants lived in housing areas (n = 227, 52.4%), followed by shop houses (180, 41.6%) and squatter houses (n = 26, 6%). The overall seroprevalence of *Leptospira* infection was 24.7% [19.60–30.62%] with 3.0% [1.42–6.07%] seropositive for anti-*Leptospira* IgG and 21.7% [16.82–27.39%] for anti-*Leptospira* IgM.

### Risk factors and anti-*Leptospira* IgG and IgM antibodies

Few sociodemographic and lifestyle factors showed significant association with anti-*Leptospira* IgG seroprevalences. The first risk factor associated with IgG seroprevalence was age (*χ*^2^_2_ = 11.968, *P* = 0.003) with prevalence being high among participants older than 18 years of age. The second risk factor was ethnicity (*χ*^2^_5_ = 17.631, *P* = 0.003), particularly high among the Kachin and Rakhine, and pet ownership (*χ*^2^_1_ = 10.717, *P* = 0.001), with higher values amongst owners who did not have pets (Table 1).

**Table 1:** Potential risk factors on socio-demographic and lifestyle factors associated with IgG + seropositivity of *Leptospira* infections in the refugee population (univariate analysis; N = 433); *significant at 0.05

Factors	%	95% CL	OR (95% CI)	P-value
Socio-demographic factors				
Sex				
Male (n = 223)	3.6	2.2–5.7	1.526 (0.491, 4.740)	0.460
Female (n = 210)	2.4	1.3–4.2	1.000	
Age*				
> 18 (n = 144)	5.6	2.8–10.6	1.565 (0.499, 4.905)	**0.003**
12–18 (n = 138)	3.6	1.5–8.0	1.000	
< 12 (n = 151)	0.0	0.0–2.8		
Ethnicity*				
Kachin (n = 185)	6.5	3.1–12.8	4.301 (0.548, 33.761)	**0.003**
Rakhine (n = 63)	1.6	0.2–7.4	1.000	
Rohingya (n = 107)	0.0	0.0–2.0		
Syrian (n = 19)	0.0	0.0–17.6		
Pakistan (n = 34)	0.0	0.0–8.2		
Chin (n = 25)	0.0	0.0–13.4		
Education attainment				
Yes (n = 395)	3.3	1.7–6.2		0.297
No (n = 38)	0.0	0.0–9.0		
Accommodation type				
Shop houses (n = 180)	3.3	1.1–8.5	1.084 (0.358, 3.283)	0.437
Housing area (n = 227)	3.1	1.8–5.1	1.000	
Squatter (n = 26)	0.0	0.0–12.9		
Lifestyle factors				
Eating habits by hand				
No (n = 152)	4.6	2.0–9.6	2.213 (0.730, 6.706)	0.161
Yes (n = 281)	2.1	1.1–4.2	1.000	
Drinking water sources				
Boil (n = 166)	3.6	1.3–8.6	1.393 (0.460, 4.218)	0.560
Filter (n = 267)	2.6	1.4–4.8	1.000	
Proper waste disposal area				
Yes (n = 359)	3.6	1.9–6.5		0.201
No (n = 74)	0.0	0.0–5.5		
Frequency of hand washing				
> 5 times (n = 226)	3.5	2.2–5.7	1.196 (0.384, 3.724)	0.621
3–5 (n = 168)	3.0	1.0–7.7	1.000	
< 3 times (n = 39)	0.0	0.0–9.1		
Pet ownership*				
No (n = 179)	6.1	2.9–12.1	8.250 (1.806, 37.693)	**0.001**
Yes (n = 254)	0.8	0.3–2.2	1.000	

*Significance values from Log-Linear Models

Analyses for anti-*Leptospira* IgM on the other hand identified several other significant risk factors. The first risk factor associated with IgM seroprevalence was sex (*χ*^2^_1_ = 7.115, *P* = 0.008), with higher prevalence among females compared with males. Ethnicity (*χ*^2^_5_ = 39.033, *P* < 0.00001) also showed significant association with anti-*Leptospira* IgM, especially amongst Rohingyas and Pakistani. Respondents who ate using hands (*χ*^2^_1_ = 10.713_,_ *P* = 0.001) and had pets (*χ*^2^_1_ = 8.140_,_ *P* = 0.004) (Table 2) were also positively associated with anti-*Leptospira* IgM.

**Table 2:** Potential risk factors on socio-demographic and lifestyle factors associated with IgM + seropositivity of *Leptospira* infections in the refugee population (univariate analysis; N = 433); *significant at 0.05

Factors	%	95% CL	OR (95% CI)	P-value
Socio-demographic factors				
Sex*				
Female (n = 210)	27.1	23.3–31.3	1.873 (1.175, 2.984)	**0.008**
Male (n = 223)	16.6	13.4–20.3	1.000	
Age				
< 12 (n = 151)	20.5	14.4–28.1	1.172 (0.657, 2.094)	0.191
12–18 (n = 138)	26.8	20.1–34.5	1.663 (0.943, 2.933)	
> 18 (n = 144)	18.1	12.4–25.3	1.000	
Ethnicity*				
Rohingya (n = 107)	40.2	33.2–47.4	4.507 (2.530, 8.028)	** < 0.00001**
Pakistan (n = 34)	29.4	17.7–44.2	2.795 (1.191, 6.561)	
Chin (n = 25)	24.0	11.0–43.9	2.118 (0.769, 5.834)	
Rakhine (n = 63)	17.5	10.4–27.4	1.419 (0.651, 3.093)	
Kachin (n = 185)	13.0	7.5–20.5	1.000	
Syrian (n = 19)	0.0	0.0–17.6		
Education attainment				
No (n = 38)	31.6	18.7–47.3	1.762 (0.852, 3.641)	0.138
Yes (n = 395)	20.8	16.2–26.1	1.000	
Accommodation type				
Squatter (n = 26)	23.1	10.6–42.2	1.159 (0.435, 3.093)	0.884
Housing area (n = 227)	22.5	18.8–26.6	1.120 (0.695, 1.805)	
Shop houses (n = 180)	20.6	13.8–28.9	1.000	
Lifestyle factors				
Eating by hand*				
Yes (n = 281)	26.3	22.0–31.1	2.359 (1.375, 4.049)	**0.001**
No (n = 152)	13.2	8.2–19.9	1.000	
Drinking water sources				
Filter (n = 267)	24.7	20.7–29.3	1.618 (0.989, 2.648)	0.051
Boil (n = 166)	16.9	11.1–24.7	1.000	
Proper waste disposal area				
Yes (n = 359)	22.6	18.1–27.8	1.367 (0.715, 2.613)	0.868
No (n = 74)	17.6	10.1–28.4	1.000	
Frequency of handwashing				
< 3 times (n = 39)	20.5	10.3–36.3	1.010 (0.435, 2.344)	0.161
3–5 (n = 168)	23.8	16.9–32.3	1.223 (0.756, 1.977)	
> 5 times (n = 226)	20.4	16.8–24.3	1.000	
Pet ownership*				
Yes (n = 254)	26.4	22.3–30.9	2.017 (1.229, 3.310)	**0.004**
No (n = 179)	15.1	9.3–22.9	1.000	

*Significance values from Log-Linear Models

The presence of anti-*Leptospira* IgG showed significant association with a basic understanding of leptospirosis by refugees (*χ*^2^_1_ = 6.967_,_ *P* = 0.008). Their awareness of mortality and disease (*χ*^2^_1_ = 4.283, *P* = 0.038) (Table 3) also showed significant association, although lower IgG seroprevalences were found in only a very small sample of refugee with some relevant knowledge of leptospirosis.

**Table 3:** Potential risk factors on knowledge and etiology factors associated with IgG + seropositivity of *Leptospira* infections in the refugee population (univariate analysis; N = 433); *significant at 0.05

Factors	%	95% CL	OR (95% CI)	P-value
Prior knowledge on leptospirosis				
Basic knowledge*				
Yes (n = 30)	13.3	4.7–29.8	6.735 (1.943, 23.341)	**0.008**
No (n = 403)	2.2	1.0–5.0	1.000	
Disease-related mortality*				
Yes (n = 26)	11.5	3.2–30.4	5.178 (1.333, 20.115)	**0.038**
No (n = 407)	2.5	1.1–5.3	1.000	
Transmission by rats				
Yes (n = 37)	5.4	1.2–17.3	2.000 (0.426, 9.384)	0.414
No (n = 396)	2.8	1.3–5.6	1.000	
Etiological factors				
Presence of rats				
No (n = 151)	4.0	1.6–8.8	1.626 (0.536, 4.927)	0.395
Yes (n = 282)	2.5	1.3–4.7	1.000	
Contact with rat urine				
Yes (n = 56)	3.6	1.1–9.7	1.232 (0.266, 5.711)	0.794
No (n = 377)	2.9	1.5–5.7	1.000	
Walking bare feet				
No (n = 296)	4.1	2.4–6.7	5.746 (0.740, 44.649)	0.496
Yes (n = 137)	0.7	0.1–3.8	1.000	
Involvement in flooding				
Yes (n = 65)	4.6	1.5–11.9	1.732 (0.464, 6.472)	0.436
No (n = 368)	2.7	1.3–5.4	1.000	
Water recreation visits				
Yes (n = 139)	3.6	1.5–8.0	1.334 (0.428, 4.155)	0.623
No (n = 294)	2.7	1.4–5.0	1.000	

*Significance values from Log-Linear Models

However, the occurrence of anti-*Leptospira* IgM was associated with the presence of rats in the community (*χ*^2^_1_ = 7.300_,_ *P* = 0.007). Two risk factors; walking in bare feet (*χ*^2^_1_ = 4.160, *P* = 0.041) and visiting water recreation sites (*χ*^2^_1_ = 4.711, *P* = 0.030) also showed significant association with anti-*Leptospira* IgM seroprevalence (Table 4).

**Table 4:** Potential risk factors on knowledge and etiology factors associated with IgM + seropositivity of *Leptospira* infections in the refugee population (N = 433); *significant at 0.05

Factors	%	95% CL	OR (95% CI)	P-value
Prior knowledge on Leptospirosis				
Basic knowledge				
No (n = 403)	22.1	17.4–27.6	1.417 (0.527, 3.809)	0.475
Yes (n = 30)	16.7	6.8–34.8	1.000	
Disease-related mortality				
Yes (n = 26)	23.1	10.6–42.2	1.088 (0.424, 2.791)	0.862
No (n = 407)	21.6	16.9–27.1	1.000	
Disease by rat transmission				
No (n = 396)	22.2	17.5–27.7	1.476 (0.597, 3.652)	0.382
Yes (n = 37)	16.2	7.3–30.8	1.000	
Etiological factors				
Presence of rats*				
Yes (n = 282)	25.5	21.3–30.2	2.010 (1.189, 3.400)	**0.007**
No (n = 151)	14.6	9.4–21.6	1.000	
Contact with rat urine				
No (n = 377)	21.8	17.2–27.0	1.019 (0.514, 2.019)	0.956
Yes (n = 56)	21.4	14.0–31.1	1.000	
Walking in bare feet*				
Yes (n = 137)	27.7	21.1–35.5	1.645 (1.024, 2.642)	**0.041**
No (n = 296)	18.9	15.1–23.4	1.000	
Involvement in flooding				
Yes (n = 65)	27.7	18.7–38.5	1.471 (0.808, 2.679)	0.215
No (n = 368)	20.7	16.2–25.8	1.000	
Water recreation visits*				
Yes (n = 139)	28.1	21.3–35.9	1.695 (1.057, 2.717)	**0.030**
No (n = 294)	18.7	14.9–23.2	1.000	

*Significance values from Log-Linear Models

Symptoms of illness appeared to show that the occurrence of headaches (*P* = 0.001) and fever (*P* = 0.003) were negatively associated with leptospirosis (Table 5), since in both cases IgG seroprevalence was higher in those without these symptoms.

**Table 5:** Seroprevalences of IgG+ and IgM+ antibodies to leptospirosis relative to clinical symptoms in the refugee population; * significant at 0.05

	IgG		IgM	
	% (95% CL)	P-value	% (95% CL)	P-value
Headache*				
Yes (n = 251)	0.8 (0.3–2.2)	**0.001**	22.7 (18.9–27.0)	0.552
No (n = 182)	6.0 (2.8–12.0)		20.3 (13.6–28.8)	
Jaundice				
Yes (n = 39)	2.6 (0.2–13.6)	0.863	23.1 (12.1–38.9)	0.829
No (n = 394)	3.0 (1.5–5.9)		21.6 (16.9–26.9)	
Myalgia				
Yes (n = 176)	2.3 (0.6–7.1)	0.454	21.6 (14.9–30.1)	0.961
No (n = 257)	3.5 (2.1–5.8)		21.8 (18.0–26.1)	
Chills				
Yes (n = 125)	0.8 (0.1–3.7)	0.055	22.4 (16.5–29.5)	0.825
No (n = 308)	3.9 (2.2–6.5)		21.4 (17.3–26.1)	
Fever*				
Yes (n = 235)	0.9 (0.3–2.3)	**0.003**	21.3 (17.6–25.4)	0.812
No (n = 198)	5.6 (2.4–11.9)		22.2 (15.0–31.6)	
Diarrhea				
Yes (n = 82)	2.4 (0.4–10.0)	0.734	29.3 (19.1–41.8)	0.073
No (n = 351)	3.1 (1.6–5.8)		19.9 (15.8–24.9)	
Abdominal discomfort				
Yes (n = 143)	3.5 (1.4–7.9)	0.676	25.9 (19.2–33.6)	0.144
No (n = 290)	2.8 (1.5–5.0)		19.7 (15.8–24.1)	

*Significance values from Log-Linear Models

Multifactorial analyses of IgG seroprevalences in a model where host age, ethnicity, pet ownership, basic knowledge leptospirosis, and awareness of disease related mortality were assessed, indicated that pet ownership appeared to be the most significant factor (*χ*^2^_1_ = 5.95, *P* = 0.015). This was a robust finding with pet owners demonstrating significantly higher seroprevalences than those who did not own pets (Table 1). This analysis also revealed a second significant expression (IgG seroprevalence × basic knowledge, *χ*^2^_1_ = 5.79, *P* = 0.016) where refugees with a basic knowledge of leptospirosis showed a higher prevalence than those without such knowledge, but the sample sizes were very small (Table 3). This analysis also confirmed that age was a significant risk factor, IgG seroprevalence being significantly higher in refugees more than 18 years old (χ^2^_2_ = 7.79, *P* = 0.020).

A separate multifactorial model which was used to compare IgG seroprevalences with clinical symptoms (Table 5) and this revealed only headache with fever as significant (*χ*^2^_1_ = 8.167, *P* = 0.004). This arose because among subjects with neither fever nor headaches, IgG seroprevalence was high (10.0% [95% CI 6.31–15.19]) compared with subjects with headache, with/without fever and no headache but with fever.

**Fig. 1:**
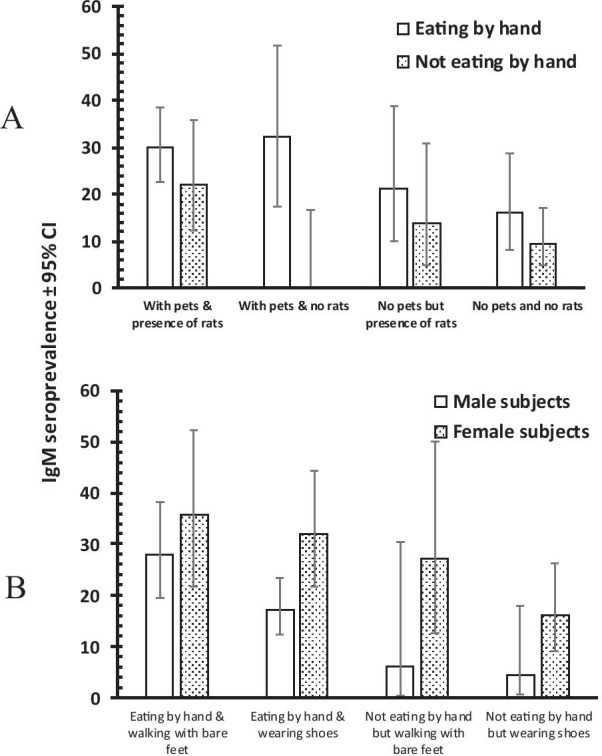
IgM seroprevalence in the refugee population (i). **A** IgM seroprevalence according to whether subjects ate by hand or with cutlery, kept pets or not, and whether rats were present in the household; this interaction using a multifactorial minimum sufficient model (MMSM) was significant (*χ*^2^_1_ = 5.761, *P* = 0.016). **B** IgM seroprevalence in male and female subjects according to whether they ate by hand or with cutlery and walked about barefoot or wore footwear (shoes) and this interaction was also significant (*χ*^2^_1_ = 5.314, *P* = 0.021)

Multifactorial analysis of IgM seroprevalences on the other hand revealed a more complex picture. We again fitted all significant factors from first stage analyses of refugees including sex, ethnicity, eating by hand, presence of pets, walking barefoot, the presence of wild rodents within communities and visiting water recreation centres. The minimum sufficient model comprised three expressions the first of which is shown in Fig. 1A (*χ*^2^_1_ = 5.761, *P* = 0.016).

In all cases, IgM seroprevalences were higher in those who ate by hand but lower in those without pets, while the presence/absence of rats in their communities made little difference. However, IgM seroprevalences were lower in those who neither ate by hand nor kept pets, even in the presence of rats, compared with higher IgM levels in communities where both pets and rats were present.

The second significant interaction (*χ*^2^_1_ = 5.314, *P* = 0.021) showed that IgM seroprevalence was higher in female compared with male subjects in all groups but lower in those who did not eat by hand and wore shoes (Fig. 1B). In male subjects the trend was similar with reduced IgM values in those who did not eat by hand, irrespective of whether they wore shoes or walked barefoot.

**Fig. 2:**
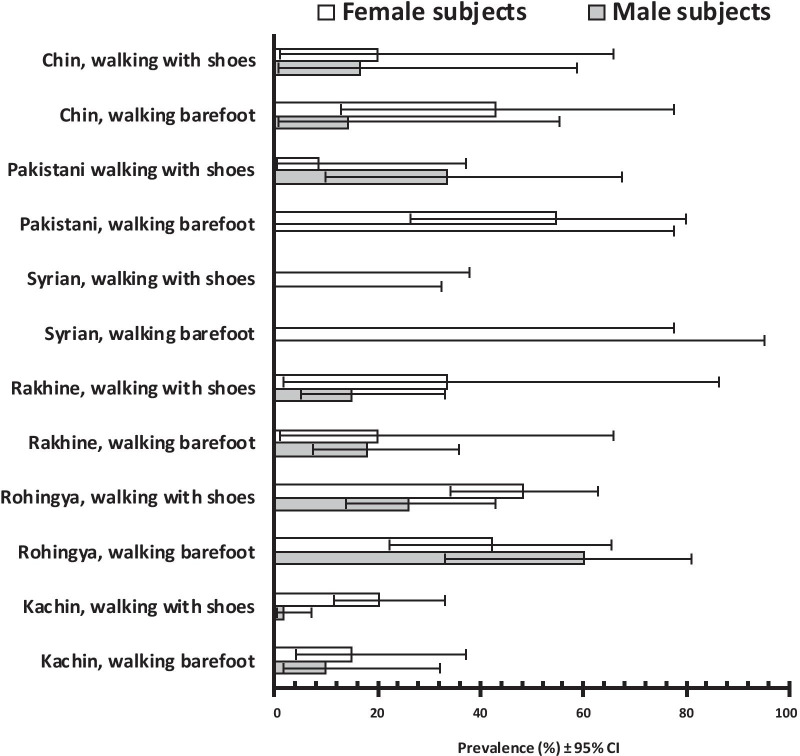
IgM seroprevalence in the refugee population (ii). IgM seroprevalence in male and female subjects, according to their ethnic backgrounds and whether they walk about barefoot or with footwear (shoes); this interaction using MMSM was significant (*χ*^2^_1_ = 5.314, *P* = 0.021)

The third significant interaction in IgM seroprevalence, relative to sex, ethnicity and walking barefoot (*χ*^2^_5_ = 14.020, *P* = 0.015), is more complex as there are 24 data subsets in six ethnic groups (Fig. 2).

IgM seroprevalences were found to be overall significantly higher in female subjects (Table 2) as shown among the Chin (n = 25), Rakhine (n = 63) and Kachin (n = 185), whether walking barefoot or wearing shoes. IgM values amongst the Rohingya (n = 107) and Pakistani subjects (n = 34) differed between the sexes, depending on whether they walked barefoot or wore footwear, whereas none among the Syrian sample (n = 19) were found to be positive.

## Discussion

In the present study a substantial proportion (24.7%) of the study cohort not only showed seropositivity for anti-*Leptospira antibodies*, but the majority (21.7% of study cohort and 88% of positive cases) were also IgM seropositive. In contrast, only a small proportion (3%) of participants were IgG seropositive, indicating previous exposure. IgG antibodies persist for many years following infection and their presence does not necessarily indicate current or active infections [19], but rather recovery from infection acquired at some earlier time. Therefore, the significant IgM seroprevalence (21.7%), particularly amongst Rohingyas and Pakistani refugees, indicated that exposure to *Leptospira* was most likely acquired after entry to Malaysia, rather than prior to leaving their country of origin.

Malaysia is not a signatory party to the 1951 Refugee Convention that relates to the Status of Refugees and its 1967 Protocol, that safeguards the fundamental rights of refugees, regulates their status, and requires their essential needs to be provided in those countries offering asylum. Therefore, there is no legal framework to register, document and recognize the status of refugees and their rights in this country. These communities are totally excluded from the government system and thus vulnerable to detention and exploitation, especially individuals without the possession of UNHCR identification cards. Many live in squatter homes and overcrowded flats with poor waste management and sanitation, conditions that create an ideal environment for rodents to thrive in. Consequently, there is an elevated risk of transmission of rodent-borne zoonotic infections such as leptospirosis. This disease is endemic in the Asian-Pacific region where poor socioeconomic, and behavioral conditions facilitate its incidence and prevalence [20] and are also responsible for mortality in the case of some Burmese refugees at the Juru Detention Centre in Penang [21, 22].

The highest IgM seroprevalence was recorded among subjects who routinely ate food by hand and owned pets, whether rats were or were not present in their living quarters, and both are clearly risk factors in the study cohort. Wild and domesticated mammals including pet cats and dogs have been implicated in the transmission of leptospirosis [23] by acting as maintenance and incidental hosts for some serovars. Both feline and canine hosts are likely to interact frequently with wild rodents thereby providing an important link in the chain of transmission and this was confirmed with presence of two pathogenic *Leptospira* serovars recovered from urine and kidney samples from 150 local stray dogs [24]. Pet owners are more likely to experience scratching of the skin during physical contact with pets, which in turn may serve as a point of entry for infectious agents, as confirmed in studies where pets were responsible for numerous severe cases of human leptospirosis [25, 26].

There was also a strong sex-linked effect on IgM seroprevalence, which was higher among female, compared with male subjects, whether or not they ate by hand or utilized footwear. The female bias in IgM seroprevalence is consistent with studies on hospital patients [27–29], whereas conversely Kawaguchi et al. found higher levels of infection in male hosts [30]. Leptospirosis infections are more likely to take place during outdoor activities through contact with soil and water contaminated with animal urine [8]. It is possible that more females are involved in outdoor activities where rodent populations are high, and when combined with eating by hand (Fig. 1B) this may explain to some extent female bias in IgM seroprevalence. Therefore, sex bias in the incidence and prevalence of leptospirosis among deprived populations is more likely explained by differences in environmental, recreational and travel-related activities between sexes rather than being directly attributable to host sex per se [31].

Age was initially identified as a risk factor for IgG seroprevalence in our preliminary analyses (Table 1), and also when other factors had been taken into account in multifactorial models, but statistical analysis failed to identify age as a risk factor for IgM seroprevalence. Nevertheless, there was some indication of higher IgM seroprevalence among teenage children, who are likely to be the most active sector of any population. Moreover, if they do not wear footwear, they are likely to make direct or indirect contact with contaminated urine, carcasses of infected animals and the reservoir hosts. Based on our observations, both the Rohingya and Pakistani communities commonly lives in buildings associated with bad sanitation and poor waste management which allow the rat populations to thrive, thus contributing to the risk of contracting leptospirosis [32, 33].

Indeed, the presence of rats in living quarters was identified as a risk factor for IgM seroprevalence in the initial preliminary statistical models, and subsequently in the multifactorial model, as a component of the interaction with pet ownership and method of eating food. Values for IgM seroprevalence were marginally higher among those who lived in accommodation where rats were present. Overcrowding and poverty have been implicated in rodent-borne transmission of leptospirosis in Bangladesh [34]. Furthermore, a recent study by Sahimin et al. reported the seroprevalence of anti-*Leptospira* IgG and IgM antibodies amongst urban residents of low-cost flats in Kuala Lumpur [15] whereas Benacer et al. identified two pathogenic *Leptospira* serovars; *L. borgpetersenii* serovar Javanica and *L. interrogans* serovars Bataviae in urban rat populations in Peninsular Malaysia [35]. These studies, together with the present, therefore highlight poor hygiene practices, inadequate sanitation and the presence of rodents as major risks for leptospirosis infections in Malaysian urban communities [15].

Eating style was another risk factor associated with the raised IgM in the current study. Hand hygiene is known to be important as transmission of leptospirosis can take place through skin penetration [8]. This is particularly relevant for those involved in water recreational water activities, which constitute an important risk factor for the transmission of leptospirosis. Many articles have reported that following exposure to contaminated water and soil during recreational activities, such as jungle hiking, water rafting, swimming and other related water activities, there is an increased risk of acquiring leptospirosis [36–39]. Water recreation visits were initially found to be associated with anti-*Leptospira* IgM in the current work, but not when other factors had not been taken into account in the multifactorial analysis. Nevertheless, as reported elsewhere, outbreaks of leptospirosis related to water recreational activities have shown the capacity of pathogenic *Leptospira* species to live in water for prolonged periods of time, thereby increasing the possibility of infecting a susceptible host [40]. This aquatic route of transmission therefore represents indirect transmission of leptospirosis from animals to humans.

Clinical symptoms often associated with leptospirosis include headaches, fever, jaundice, chills, muscle pain or myalgia, abdominal discomfort, and diarrhea, but in the refugee community from the Klang Valley, none of these symptoms were associated with anti-*Leptospira* IgG and IgM. Multifactorial models correlating clinical symptoms with IgG seroprevalences showed that refugees without fevers or headaches had higher levels of IgG. The latter were more likely to be due to past acute infections with IgG dependent immunity still being expressed. On the other hand, leptospirosis can also be asymptomatic especially in areas with high transmission rates [41], resulting in the disease being critical in later stages when kidneys, lungs and the heart cease to function [42]. Infection with *Leptospira* can typically manifest itself in a range of nonspecific clinical symptoms such as acute febrile illness with fever, myalgia, arthralgia and headaches [43–45]. Consequently, the infection is frequently misdiagnosed and underreported [46, 47] especially as symptoms also mimic influenza and dengue fever [48–51]. In more severe cases hemorrhages and multi-organ failure can occur and potentially can be fatal [46].

Overall, the evidence from this study highlighted that many of the participants with past leptospirosis infection most probably acquire the disease due to bad living environmental condition and their lifestyle behaviour that have exposed them to contaminated urine from infected animal reservoirs. This study also suggests that most of the participants may only have limited knowledge on the transmission of the disease.

Moving forward there is a need to look at approaches in delivering awareness of disease transmission to the community to prevent the occurrence leptospirosis outbreak. This can be made through the empowerment of the community via community engagement activities such as wellness program and clean-up activities. However, the delivery of the information must be in their respective languages to enhance better understanding.

Nevertheless, there may be some biases as there were several limitations to the study. Firstly, this study was conducted with a specific cohort group i.e., students and secondly this screening was conducted as a cross sectional study thus, not able to represent the whole refugee community.

## Conclusions

The substantial (24.7%) seroprevalence of leptospirosis infection amongst students in the refugee community warrants an introduction to proper environmental sanitation and good lifestyle behavior programs in tandem with improved knowledge on disease transmission through community engagement activities. Although the prevalence of *Leptospira* infection was not assessed in the current study, it is known to be a rodent borne infection with rats being major reservoirs of this and other zoonotic infections. Therefore, a much-improved rodent control program should therefore be undertaken in these low cost residential infrastructures where many of these communities reside.

## Data Availability

All data generated or analysed during this study are included in this article.

## Abbreviations

UNHCR: United Nations High Commissioner for Refugees
IgG: Immunoglobulin G
IgM: Immunoglobulin M
ELISA: Enzyme-linked immunosorbant assay
OD: Optical densities
CFT: Complement fixation test
U/ml: Units per milliliter
ml: Milliliter
RPM: Revolutions per minute
nm: Nanometer
SPSS: Statistical Package for the Social Sciences
CL: Confidence limits
CI: Confidence interval
MSSM: Multifactorial minimum sufficient model
HEI: Health Equity Initiatives
*P*-value: Probability value
N: Population size
